# Combined Preoperative Risk Score Including sCD40, Leukocytes, and BMI Predicts Pancreas-Specific Complications After Pancreatic Cancer Surgery

**DOI:** 10.1245/s10434-026-19628-8

**Published:** 2026-05-02

**Authors:** Loreen Natusch Bufe, Marie Crede, David Digomann, Antonia M. A. König, Anna Klimova, Tido Willms, Carolin Beer, Sarah Cronjaeger, Sebastian Hempel, Florian Oehme, Michael Ghadimi, Florian Bösch, Jürgen Weitz, Adrian M. Seifert, Lena Seifert

**Affiliations:** 1 Department of Visceral, Thoracic and Vascular Surgery, Medical Faculty, University Hospital Carl Gustav Carus, Technische Universität Dresden, Dresden, Germany; 2 Medizinische Fakultät Carl Gustav Carus Dresden, Technische Universität Dresden, Dresden, Germany; 3 National Center for Tumor Diseases (NCT), University Hospital Carl Gustav Carus, Technische Universität Dresden, Dresden, Germany; 4 German Cancer Research Center (DKFZ), German Cancer Consortium (DKTK), Heidelberg, Germany; 5 Department of Surgery, University Medical Center, Göttingen, Germany; 6 Else Kröner Clinician Scientist Professor for Translational Tumor Immunological Research, Technische Universität Dresden, Dresden, Germany

**Keywords:** Soluble CD40, PDAC, Pancreas-specific complications, POPF, PPH

## Abstract

**Background:**

Pancreatic ductal adenocarcinoma (PDAC) has a poor prognosis, with a 5-year survival rate of 13%. Surgical resection followed by adjuvant chemotherapy remains the only curative approach. However, complications such as postoperative pancreatic fistula (POPF) and postpancreatectomy hemorrhage (PPH) often delay or prevent further treatment. Reliable preoperative biomarkers for predicting these complications are lacking. This study investigated soluble CD40 (sCD40) as a potential predictive marker for pancreas-specific complications after pancreatoduodenectomy (PD) in patients with PDAC.

**Methods:**

Preoperative serum samples from 185 patients with PDAC undergoing pylorus-preserving pancreatoduodenectomy or a Whipple procedure were analyzed using enzyme-linked immunosorbent assay to quantify sCD40 levels. Clinical and postoperative data were systematically collected and classified.

**Results:**

Of the 185 patients, 151 underwent pylorus-preserving PD and 34 a Whipple procedure. Clinically relevant POPF occurred in 9.7% and PPH in 7.6% of patients. Preoperative sCD40 levels were significantly lower in patients who developed POPF or PPH (*P* = 0.025 and *P* = 0.008). The association remained significant in multivariable analysis. Receiver operating characteristic analysis demonstrated an area under the curve of 0.660 for sCD40. Adding leukocytes and body mass index improved predictive performance (area under the curve 0.705 for POPF and 0.752 for PPH).

**Conclusion:**

Reduced preoperative sCD40 serum levels are associated with a higher risk of POPF and PPH after PD. Combining sCD40 with leukocytes and BMI may enhance preoperative risk assessment in patients with PDAC.

**Supplementary Information:**

The online version contains supplementary material available at 10.1245/s10434-026-19628-8.

With a 5-year overall survival rate of only 13%, pancreatic ductal adenocarcinoma (PDAC) has a dismal prognosis and is predicted to become the second leading cause of cancer-related deaths in the USA by 2030.^1,2^ This poor prognosis is driven by an immunosuppressive tumor microenvironment and an aggressive tumor biology. Patients are typically asymptomatic in the early stages of the disease and so are often diagnosed in an advanced stage, and more than half of them have metastatic disease at the time of diagnosis.^1^ Currently, the only curative treatment is surgical resection of the tumor. The most frequently performed surgical procedures are pancreatoduodenectomy (PD), followed by left pancreatectomy and total pancreatectomy.^3–6^ In the field of pancreatic surgery, postoperative pancreatic fistula (POPF) remains the major cause of morbidity and mortality. POPF, defined as leakage of exocrine pancreatic secretion, is one of the most threatening procedure-specific complications after PD,^7^ in severe cases followed by postpancreatectomy hemorrhage (PPH). The International Study Group of Pancreatic Surgery established a precise definition for clinically relevant POPF (CR-POPF), which is described as a postoperative drain output with a three times increased amylase level compared with the upper serum amylase level and is associated with an impaired clinical condition.^7,8^ In terms of their clinical impact, POPF is classified as biochemical leak (increased amylase without clinical impact), POPF grade B (associated with treatment modifications because of the fistula), and POPF grade C (postoperative fistula with life-threatening complications, reoperation, and intensive care treatment).^7^ The score most commonly used to predict the risk of developing a POPF after surgery is the fistula risk score (FRS), which includes pathology, intraoperative blood loss, pancreatic texture, and duct diameter.^9^ However, to inform patients and potentially consider neoadjuvant therapy, risk prediction must be available before surgery to enable better shared decision-making and treatment optimization. Further, POPF is significantly associated with PPH, one of the most common causes of intervention and reoperation after PD.^10^ PPH is classified into three grades (A–C) based on timing, location, and severity and ranging from mild early bleeding requiring observation (grade A) to moderate bleeding requiring transfusion or endoscopic treatment (grade B) and severe, life-threatening bleeding necessitating intensive care and intervention (grade C). Depending on the bleeding site, urgent interventions such as angiographic embolization or relaparotomy are often necessary to control the bleeding.^11,12^ To date, no reliable biomarker exists for preoperative POPF or PPH risk prediction. CD40, a transmembrane receptor to the tumor necrosis factor superfamily, plays a key role in humoral and adaptive immunity and is mainly expressed on antigen-presenting cells.^13,14^ Currently, CD40 is primarily studied in the context of cancer biology and immune regulation. Previously, we have identified soluble CD40 (sCD40) as a diagnostic and prognostic biomarker for PDAC.^15^ To date, no studies have investigated the role of sCD40 for risk prediction of POPF and PPH.

## Patients and Methods

### Study Design and Sample Collection

The study included serum samples from 135 patients with PDAC who underwent PD or pylorus-preserving PD (PPPD) at the Department of Visceral, Thoracic and Vascular Surgery at the University Hospital Carl Gustav Carus Dresden and from 50 patients with PDAC who underwent surgical resection at the Clinic of General, Vascular and Pediatric Surgery, University Medical Center, Göttingen. Venous blood was either obtained immediately before surgery or up to 10 days before surgery and drawn into serum separator tubes. All samples were centrifuged immediately and stored at −80°C. Samples were collected between 2006 and 2022. The pancreatic anastomosis was performed as a double-layer duct-to-mucosa pancreatojejunostomy with an additional outer seromuscular layer of sutures. This technique represents the standard approach in both institutions. All patients gave informed consent, and the protocol was approved by the ethics committee of the Technische Universität Dresden (No. EK446112017). Pancreas-specific postoperative complications were identified and diagnosed in accordance with the standardized clinical and radiological criteria established by the International Study Group of Pancreatic Surgery.^7,11,12^

### Enzyme-linked Immunosorbent Assay

For the detection of sCD40, enzyme-linked immunosorbent assay (ELISA) was used according to the manufacturer’s instructions (Human CD40 Quantikine ELISA Kit, Catalog #: DCCD40, R&D Systems, Minneapolis, USA). Varioskan LUX (Thermo Fisher, Waltham, MA, USA) was used for detection. All experiments were performed at the laboratory of the Department of Visceral, Thoracic and Vascular Surgery at the University Hospital Carl Gustav Carus Dresden. Leukocytes, thrombocytes, and tumor marker carbohydrate antigen 19-9 (CA 19-9) were determined via the clinical laboratory.

### Statistical Analysis

Data are presented with the median in dot plots. The Mann–Whitney *U* test was applied for grouped analysis. If patient numbers in a certain group were low, Fisher’s t-test was applied. Predictive values of single parameters were evaluated by receiver operating characteristic (ROC) curves with area under the curve (AUC). *P*-values ≤ 0.05 were considered statistically significant, and GraphPad Prism 10.4.1 (GraphPad Software, San Diego, CA, USA) was used for analysis. Multivariable logistic regression was used to assess the predictive value of the combined parameters, using SPSS for statistical analysis (SPSS Statistics for Windows, version 30.0; IBM Corp., Armonk, NY, USA).

## Results

**Fig. 1:**
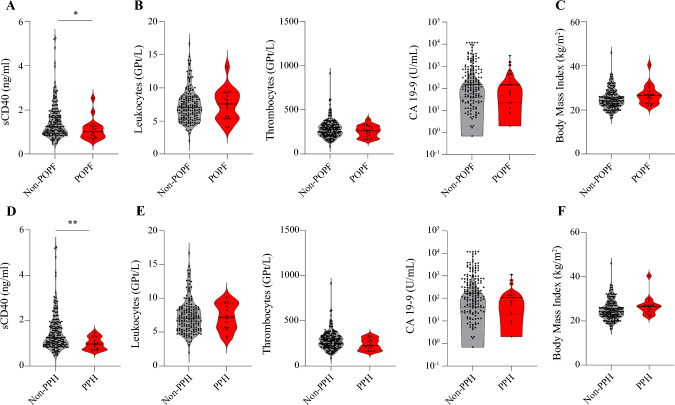
Soluble CD40 (sCD40), additional blood parameters, and body mass index in patients with postoperative pancreatic fistula (POPF) and postpancreatectomy hemorrhage (PPH). **A** sCD40, **B** leukocytes, thrombocytes, carbohydrate antigen (CA) 19-9, and **C** body mass index in patients with pancreatic ductal adenocarcinoma (PDAC) measured before surgery who developed a POPF. **D** Serum levels of sCD40, **E** leukocytes, and **F** body mass index measured preoperatively in patients with PDAC who developed PPH compared with non-PPH controls. Statistical analysis was performed using the Mann–Whitney *U* test. Each data point represents an individual patient, and the black line indicates the median. *P < 0.05, **P < 0.01

**Fig. 2:**
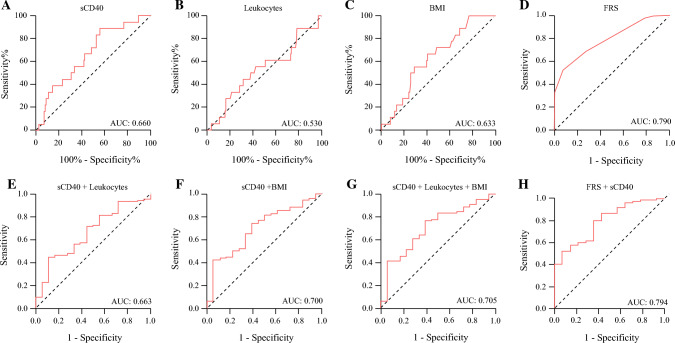
Predictive value of soluble CD40 (sCD40), leukocytes, and body mass index (BMI) for postoperative pancreatic fistula (POPF). Predictive values of sCD40, leukocytes, BMI, and Fistula Risk Score (FRS) evaluated by receiver operating characteristic (ROC) curves with area under the curve (AUC). ROC of **A** sCD40, **B** leukocytes, **C** BMI, and **D** FRS for patients with and without POPF. **E-H** ROC of sCD40, leukocytes, BMI, and FRS combined based on logistic regression for POPF

**Fig. 3:**
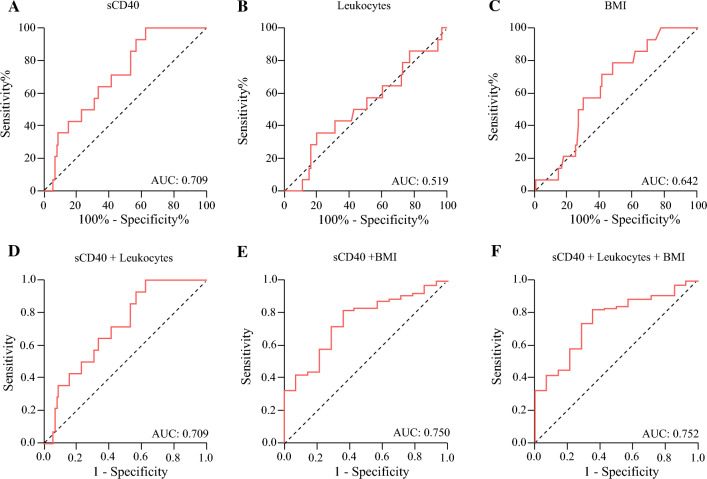
Predictive value of soluble CD40 (sCD40), leukocytes, and body mass index (BMI) for postpancreatectomy hemorrhage (PPH). Predictive values of sCD40, leukocytes, and BMI evaluated by receiver operating characteristic (ROC) curves with area under the curve (AUC). ROC of **A** sCD40, **B** leukocytes, and **C** BMI for patients with and without PPH. **D-F** ROC of sCD40, leukocytes, and BMI combined based on logistic regression for PPH

Preoperative serum levels of sCD40 were quantified using ELISA in patients with PDAC before PPPD or Whipple procedure. Additionally, leukocytes, thrombocytes, and CA 19-9 levels were determined. Of 185 patients with PDAC, 18.4% underwent a Whipple and 81.6% a PPPD. First, we examined this cohort for postoperative complications, focusing on pancreas-specific complications, especially clinically relevant POPF and PPH. Within the cohort, 18 patients (9.7%) developed a CR-POPF and 14 patients (7.6%) a CR-PPH after surgery (Supplementary Table S1). Of 18 patients with POPF, 11 developed POPF grade B (5.9%) and seven POPF grade C (3.8%). Within the 14 patients with CR-PPH, seven had PPH grade B (3.8%), and seven had grade C (3.8%). All postoperative complications were classified by Clavien–Dindo grade (Supplementary Table S2). To evaluate the potential effect of clinicopathological characteristics on sCD40 serum levels, the impact of sex, age, body mass index (BMI), diabetes mellitus, alcohol consumption, smoking, and neoadjuvant chemotherapy was analyzed (Supplementary Table S3). Next, we evaluated the predictive value of sCD40, leukocytes, thrombocytes, CA 19-9, and BMI before pancreatoduodenectomy for pancreas-specific complications. Patients who developed a POPF after pancreatoduodenectomy had significantly lower preoperative sCD40 serum levels (median 1.015) than did those without complications (median 1.250, *P* = 0.025, Fig. 1A). In contrast, other preoperative blood parameters, including leukocytes and thrombocytes, showed no significant differences between the POPF and complication-free control group, suggesting that sCD40 may serve as potential preoperative biomarker for POPF risk prediction (Fig. 1B). Given that BMI has been reported as a potential predictor of POPF in previous studies [16-18], we compared BMI between patients with POPF and complication-free controls. Unexpectedly, BMI did not differ significantly between the two groups (Fig. 1C). As PPH frequently occurs as a secondary complication of POPF, we also investigated predictors of PPH. Therefore, serum levels of sCD40, leukocytes, thrombocytes, and CA 19-9 of patients who developed a PPH after surgery were compared with those from the complication-free control group. Our results revealed a significantly lower sCD40 serum level in patients with PPH (median 0.968) than in patients with PDAC without pancreas-associated bleeding after surgery (median 1.250, *P* = 0.008, Fig. 1D). The preoperatively measured serum levels of leukocytes and thrombocytes showed no differences between both groups (Fig. 1E). Furthermore, preoperatively determined BMI showed a trend to a higher BMI in patients with PPH than in complication-free controls but without significance (Fig. 1F). Results from the univariable analysis of multiple clinicopathological parameters (age, BMI, sex, diabetes, alcohol consumption, smoking, Union for International Cancer Control (UICC) stage and neoadjuvant chemotherapy) regarding their predictive value for POPF and PPH are shown in Table 1. Patients with POPF seemed to be significantly younger than those in the complication-free control group (*P* = 0.007). Comparing patients who developed a CR-PPH and those in the control group, we found that 11 of 14 patients with CR-PPH were male (*P* = 0.003), suggesting that male patients have a higher risk of developing pancreas-associated bleeding after surgery. As a next step, we assessed the robustness of sCD40 as a potential predictive marker for POPF and PPH by constructing multivariable logistic regression models that additionally included BMI and preoperative leukocyte count. Our results demonstrate sCD40 as an independent predictive marker for the occurrence of POPF, retaining its significance (odds ratio [OR] 0.32; 95% confidence interval [CI] 0.11–0.96, *P* = 0.042) even after adjustment for BMI (OR 1.10; 95% CI 1.00–1.22, *P* = 0.062) and leukocyte count (OR 1.03; 95% CI 0.87–1.22, *P* = 0.756) in a multivariable logistic regression model. These findings affirm the biomarker's robust, independent association with POPF risk beyond established clinical factors. Furthermore, we used the same model to evaluate the predictive value of sCD40 for PPH. Although BMI (OR 1.10; 95% CI 0.98–1.24,* P* = 0.094) and leukocyte count (OR 1.01; 95% CI 0.81–1.26,* P* = 0.932) did not reach statistical significance, sCD40 remained a valid predictive biomarker for PPH (OR 0.15; 95% CI 0.03–0.71,* P* = 0.016) (Table 2). To further evaluate the predictive performance of these parameters, we conducted ROC analyses. ROC curves and the corresponding AUCs were calculated for each individual predictor to assess their discrimination ability. sCD40 exhibited a predictive ability with an AUC of 0.660, whereas leukocytes showed an AUC of 0.530, and BMI was represented with an AUC of 0.633 (Fig. 2A-C). Since FRS data were available for only a subset of patients (N = 129), ROC analysis for the FRS was performed in this subgroup, yielding an AUC of 0.790 (Fig. 2D). To determine the potential added value of combining multiple potential biomarkers, we applied a multiple logistic regression analysis integrating sCD40 and leukocyte levels as well as the BMI as predictive markers for POPF. Our results demonstrate that the combined model improves predictive accuracy, achieving an AUC of 0.705 by combining all three preoperative markers (Fig. 2E–G). Moreover, we performed multiple logistic regression analysis, including sCD40 and FRS (N = 129), yielding an AUC of 0.794 (Fig. 2H), suggesting only a minimal improvement in predictive accuracy when combining FRS with sCD40. In addition, multivariate analysis of sCD40 and the intraoperatively determined FRS was performed. Although sCD40 (OR 0.531; 95% CI 0.179–1.577, *P* = 0.254) did not reach statistical significance in this small subset, the FRS (OR 2.007; 95% CI 1.344–2.997, *P* < 0.001) remained a valid predictive biomarker (Supplementary Table S4). Furthermore, we examined the predictive value of sCD40, leukocytes, and BMI for PPH in patients with PDAC after surgery. By performing ROC analysis, sCD40 reached an AUC of 0.709, whereas leukocytes showed an AUC of 0.519, and BMI had an AUC of 0.642 (Fig. 3A–C). Combining the parameters and performing a multiple logistic regression improved the predictive accuracy (Fig. 3D, E). An AUC of 0.752 was shown, combining sCD40, leukocytes, and BMI (Fig. 3F).

**Table 1:** Univariable analysis for patients by selected pancreas-specific complications

Variable	Non-POPF (n = 167)	POPF (n = 18)	*P*-value
Median age, years	69.0	64.6	0.007** ^a^
Mean BMI, kg/m^2^	23.9	25.6	0.063 ^a^
Male sex	72 (43.1)	11 (61.1)	0.212 ^b^
Preoperative conditions			
Diabetes	66 (39.5)	5 (27.8)	0.446 ^b^
Regular alcohol consumption	9 (5.4)	4 (22.2)	0.026* ^b^
Smoking	28 (16.8)	6 (33.3)	0.107 ^b^
UICC			
I	36 (21.6)	5 (27.8)	0.555 ^b^
II	81 (48.5)	5 (27.8)	0.135 ^b^
III	40 (24.0)	6 (33.3)	0.395 ^b^
IV	10 (6.0)	2 (11.1)	0.329 ^b^
Neoadjuvant chemotherapy	21 (12.6)	3 (16.7)	0.709 ^b^

Variable	Non-PPH (n = 171)	PPH (n = 14)	*P*-value
Median age, years	68.5	65.0	0.065 ^a^
Mean BMI, kg/m^2^	25.0	26.9	0.079 ^a^
Male sex	61 (35.7)	11 (78.6)	0.003** ^b^
Preoperative conditions			
Diabetes	66 (38.6)	4 (28.6)	0.573 ^b^
Regular alcohol consumption	10 (5.8)	3 (21.4)	0.063 ^b^
Smoking	28 (16.4)	5 (35.7)	0.137 ^b^
UICC			
I	35 (20.5)	6 (42.9)	0.087 ^b^
II	82 (48.0)	4 (28.6)	0.265 ^b^
III	43 (25.1)	3 (21.4)	>0.999 ^b^
IV	11 (6.4)	1 (7.1)	>0.999 ^b^
Neoadjuvant chemotherapy	20 (11.7)	4 (28.6)	0.089 ^b^

^a^ Mann–Whitney *U* Test. ^b^ Fisher’s exact t-test. *p* < 0.05 was considered statistically significant. BMI, body mass index; POPF, postoperative pancreatic fistula, PPH, postpancreatectomy hemorrhage; UICC, Union for International Cancer Control

**Table 2:** Multivariable analysis for patients by selected pancreas-specific complications

Variable	OR	95% CI	*P*
*POPF*			
sCD40	0.319	0.106–0.962	0.042*
BMI	1.100	0.995–1.215	0.062
Leukocytes	1.027	0.866–1.219	0.756
*PPH*			
sCD40	0.149	0.032–0.705	0.016*
BMI	1.104	0.983–1.239	0.094
Leukocytes	1.010	0.809–1.260	0.932

BMI, body mass index; CI, confidence interval; OR, odds ratio; POPF, postoperative pancreatic fistula, PPH, postpancreatectomy hemorrhage; sCD40, soluble CD40.

## Discussion

Although neoadjuvant and adjuvant treatment have advanced, pancreatic surgery combined with systemic treatment remains the cornerstone of curative therapy for patients with PDAC. Although the surgical procedure is standardized, morbidity and mortality remain high. In particular, pancreas-associated complications such as POPF and PPH lead to a prolonged hospital stay and a delay in adjuvant treatment. POPF is one of the most common complications after PD and occurs in up to 30% of patients.^19^ CR-POPF is associated with a higher risk of reoperation and further complications, leading to a delay in adjuvant chemotherapy in patients with PDAC.^20^ In the past, multiple POPF risk factors, such as sex, age, BMI, diabetes, or neoadjuvant treatment, have been discussed, and although several potential predictive markers have been examined, the intraoperatively determined FRS remains the gold standard to evaluate the risk of POPF.^9,16–18,21^ To date, there is no reliable biomarker for the prediction of POPF risk before surgery. Previous work introduced a computed tomography (CT)-based FRS that assessed pancreatic texture, duct size, and remnant volume preoperatively to estimate POPF risk and guide surgical planning.^22^ An automated version, AutoFRS, was later developed, using multimodal data without human annotations, and externally validated.^23^ Nonetheless, blood biomarkers present several advantages compared with preoperative CT scans. They are minimally invasive, are more cost effective, and can be repeatedly measured to monitor disease progression without exposing patients to radiation. Furthermore, biomarkers may detect molecular changes earlier than imaging techniques and provide objective, quantitative data. Lastly, they eliminate risks associated with the use of contrast agents in CT scans. Moreover, a recent study identified four perioperative risk factors for late postpancreatectomy hemorrhage and established a Hemorrhage Risk Score to predict PPH. These factors include sentinel bleeding, a drain fluid culture positive for candida species, the presence of gas within a peripancreatic fluid collection, and rim enhancement.^24^ This study evaluated multiple clinicopathologic parameters, including various blood parameters to identify potential preoperative predictors of POPF and PPH. Notably, our findings reveal an association between the immune protein sCD40 and the development of POPF and PPH after pancreatoduodenectomy in patients with PDAC. We previously identified sCD40 as a non-invasive diagnostic and prognostic biomarker in PDAC; however, its role in predicting the risk of POPF and PPH had not yet been explored.^15^ In our analysis, preoperative sCD40 serum levels were significantly lower in patients who developed CR-POPF than in complication-free controls. Similarly, patients who experienced PPH showed a marked decrease in sCD40 compared with those without PPH. These findings suggest that sCD40 may serve as a preoperative biomarker for identifying patients at increased risk of developing pancreas-specific complications after PD. Research has linked low sCD40 levels to poor outcomes in hematologic malignancies, whereas elevated sCD40 levels have been associated with shorter time to liver metastasis in rectal cancer and with active inflammation in rheumatic diseases.^25–27^ Since sCD40 is elevated in the presence of infection or inflammation and serves as potential biomarker for inflammatory responses, inflamed pancreatic tissue may also be associated with increased serum levels of sCD40. The inflammation can promote fibrotic remodeling, resulting in a firmer pancreatic texture, which is a known protective factor against the development of POPF. Since recent work introduced a postoperative neutrophil-to-lymphocyte ratio as a prognostic parameter for POPF risk stratification, we also investigated preoperative leukocyte counts as a potential prognostic parameter for pancreas-specific complications.^28,29^ However, our analysis showed no significant differences in leukocytes between patients who developed POPF or PPH and those who did not. Since leukocytes can be influenced by various factors, such as general or local infection, their function as a predictive marker for pancreas-specific complications appears limited. Further, we recently evaluated the predictive value of preoperative frequency of blood T-cell subpopulations and detected no association with the occurrence of CR-POPF.^30^ By analyzing preoperatively measured thrombocyte levels, we examined their predictive value for POPF and PPH risk stratification. Given that POPF can lead to PPH and therefore lead to further interventions and reoperation, we specifically investigated whether baseline thrombocyte levels correlated with the risk of developing PPH. Although no significant differences between patients with and without PPH were detected, our results showed a trend toward a lower platelet count in patients who later developed a PPH, highlighting the importance of preoperative coagulation management. Additionally, we examined CA 19-9 as a potential predictive marker for POPF. Studies have shown that CA 19-9 can induce pancreatitis in murine models.^31^ Given the known association between inflammation, tissue remodeling, and reduced POPF risk because of the firmer pancreatic texture, we hypothesized that CA 19-9 levels may be lowered in patients with POPF. Moreover, preoperative serum CA 19-9 levels have been demonstrated as a potential predictive marker for POPF after pancreatic surgery in patients with PDAC.^32^ However, in this study, no difference in CA 19-9 levels between patients with pancreas-specific complications and the complication-free control group was determined. These findings suggest that CA 19-9 may not be a reliable biomarker for predicting POPF risk. Further, our data reveal a higher BMI in patients with POPF than in complication-free controls. However, this difference did not reach statistical significance. Since the establishment of the FRS as an intraoperatively applied score to predict POPF risk, multiple studies tried to improve the score by adding different parameters. One of these was the BMI, which led to the alternative fistula risk score (a-FRS). This score included intraoperatively determined parameters such as duct diameter, intraoperative blood loss, tissue texture, and pathology and was extended by BMI.^33^ More recently, another study introduced an updated alternative fistula risk score that included blood loss, duct diameter, pathology, tissue texture, BMI, and male sex.^18^ We did not observe a significant association between male sex and POPF in our cohort, but male patients were significantly overrepresented in the PPH group compared with in the complication-free group. By examining several potential risk factors for pancreas-specific complications, we found that sCD40 serum levels differed significantly in patients who developed POPF or PPH. In the subsequent multivariable logistic regression analysis, incorporating BMI and leukocyte count as covariates, sCD40 remained an independent and statistically robust predictor of POPF and PPH, whereas BMI and leukocyte count did not exhibit significant associations. Consequently, sCD40 may provide valuable additional information for risk stratification and might be integrated into future predictive models to identify high-risk patients for POPF or PPH preoperatively. By performing a ROC analysis, we aimed to evaluate whether combining sCD40, BMI, and leukocyte count could improve the predictive accuracy for POPF and PPH risk. When all three preoperatively determined parameters were combined, the AUC increased to 0.705 for POPF and to 0.750 for PPH, demonstrating that a multifactorial approach based on preoperatively available biomarkers combined with clinicopathological parameters may enhance risk stratification for pancreas-specific complications. Since the FRS is the most established biomarker for POPF risk stratification, the ROC analysis demonstrated an AUC of 0.790, confirming the strong predictive value. Our preoperative biomarker model, including sCD40, leukocytes, and BMI, reached an AUC of 0.705. Although the predictive accuracy of our model is slightly lower than that of the FRS, it has the advantage of being fully assessable before surgery and might allow early preoperative risk stratification and therefore tailored surgical approaches. By combining the FRS with sCD40, AUC increased only minimally (AUC 0.794), indicating that sCD40 adds limited improvement to the predictive value of FRS. However, the introduced preoperative biomarker combination (sCD40, leukocytes, BMI) may still serve as an essential preoperative score for POPF and PPH risk stratification before pancreatic surgery.

Limitations of this study include the relatively small patient cohort and the associated small numbers of patients with POPF or PPH. Future studies should aim to combine these parameters, to establish a scoring system depending on several factors for POPF risk stratification in a larger patient cohort. Although the current gold standard for POPF risk stratification assesses the risk intraoperatively, our approach allows for preoperative risk stratification. As a result, patients at higher risk of POPF could benefit from personalized drain management, tailored anastomotic techniques, intensified postoperative care, or even a modification of the surgical approach, such as a total pancreatectomy combined with islet cell auto transplantation.^34–38^ Our results suggest that sCD40 represents an independent predictor of POPF and PPH. Although both leukocytes and BMI were included as covariates in the multivariable regression model, neither showed a significant association with POPF or PPH occurrence. This indicates that sCD40 might capture specific pathophysiological processes related to POPF and PPH development that are not reflected by general clinicopathological parameters. Consequently, sCD40 may contribute valuable additional information for risk stratification and could be integrated into future predictive models to identify high-risk patients for POPF or PPH preoperatively. However, further studies with larger cohorts and external validation are essential to confirm these observations and to elucidate the biological mechanisms underlying this association.

## Supplementary Information

Below is the link to the electronic supplementary material.

Supplementary file1 (DOCX 29 KB)
