# Supplementary material for: Combined Preoperative Risk Score Including sCD40, Leukocytes, and BMI Predicts Pancreas-Specific Complications After Pancreatic Cancer Surgery
Source: Ann Surg Oncol. 2026 May 2;33(8):7493–502. doi: 10.1245/s10434-026-19628-8 (PMC13337751; doi:10.1245/s10434-026-19628-8)
Supplement: Supplementary file 1 — Supplementary file1 (DOCX 29 KB) [file 10434_2026_19628_MOESM1_ESM.docx]

**Supplementary Table S1.** Clinicopathological characteristics and postoperative complications of PDAC patients after pancreatoduodenectomy

| **Age** (years), median (i.q.r.) | 68.0 (12) |
| --- | --- |
| **Sex Ratio** (M:F) | 83:102 |
|  | **n = 185; n (%)** |
| **BMI category** |  |
| Underweight | 4 (2.1) |
| Normal weight | 95 (51.4) |
| Preobesity/Obesity | 86 (46.5) |
| **Pre-existing conditions** |  |
| Smokers | 34 (18.4) |
| Regular alcohol consumption | 14 (7.6) |
| Diabetes mellitus | 70 (37.8) |
| **Neoadjuvant chemotherapy** | 24 (13.0) |
| **Surgical procedure** |  |
| PPPD | 151 (81.6) |
| PD | 34 (18.4) |
| **Portal vein resection** |  |
| Yes | 66 (35.7) |
| No | 119 (64.3) |
| **Postoperative complications** |  |
| **Postoperative pancreatic fistula (CR-POPF)** | 18 (9.7) |
| Grade B | 11 (5.9) |
| Grade C | 7 (3.8) |
| **Postpancreatectomy hemorrhage (CR-PPH)** | 14 (7.6) |
| Grade B | 7 (3.8) |
| Grade C | 7 (3.8) |
| **Delayed gastric emptying (DGE)** | 28 (15.1) |
| Grade A | 10 (5.4) |
| Grade B | 9 (4.9) |
| Grade C | 9 (4.9) |
| **Chyle leak** | 18 (9.7) |
| **Disturbed wound healing** | 25 (13.5) |
| **Sepsis** | 3 (1.6) |

**Supplementary Table S2.** Clavien-Dindo grading of pancreas-specific complications after pancreatic surgery

| **Postoperative complications n (%)** | **Incidence by Clavien-Dindo Grade** | | | | | | |  |
| --- | --- | --- | --- | --- | --- | --- | --- | --- |
|  | **I** | **II** | **IIIa** | **IIIb** | **IVa** | **IVb** | **V** | |
| **Postoperative pancreatic fistula (POPF) 18 (9.7)** |  |  |  |  |  |  |  | |
| Grade B, n = 11 | 0 | 0 | 11 | 0 | 0 | 0 | 0 | |
| Grade C, n = 7 | 0 | 0 | 0 | 4 | 2 | 1 | 0 | |
| **Postpancreatectomy hemorrhage (PPH) 14 (7.6)** |  |  |  |  |  |  |  | |
| Grade B, n = 7 | 0 | 0 | 5 | 2 | 0 | 0 | 0 | |
| Grade C, n = 7 | 0 | 0 | 2 | 3 | 1 | 1 | 0 | |

**Supplementary Table S3.** Effect of clinicopathological characteristics on sCD40 serum levels in PDAC patients

| **Variable** | **sCD40 (ng/ml) Median (Range)** | ***P*** |
| --- | --- | --- |
| age < 68 years | 1.25 (0.47 – 5.25) | 0.803 |
| age ≥ 68 years | 1.21 (0.46 – 5.20) |  |
| female | 1.18 (0.46 – 5.20) | 0.861 |
| male | 1.25 (0.47 – 5.25) |  |
| BMI < 25 | 1.25 (0.51 – 5.25) | 0.184 |
| BMI ≥ 25 | 1.12 (0.46 – 3.25) |  |
| No diabetes mellitus | 1.20 (0.46 – 5.25) | 0.736 |
| Diabetes mellitus | 1.28 (0.46 – 2.87) |  |
| No regular alcohol consumption | 1.23 (0.46 – 5.25) | 0.700 |
| Regular alcohol consumption | 1.36 (0.74 – 2.37) |  |
| Non-Smokers | 1.25 (0.46 – 5.25) | 0.193 |
| Smokers | 1.19 (0.46 – 3.98) |  |
| Neoadjuvant chemotherapy | 1.14 (0.51 – 2.58) | 0.053 |
| Primary resection | 1.07 (0.46 – 5.25) |  |

**Supplementary Table S4.** Multivariable analysis for patients by selected pancreas-specific complications

| **POPF** | | | |
| --- | --- | --- | --- |
| **Variable** | **OR** | **95% CI** | ***P*** |
| sCD40 | 0.531 | 0.179 – 1.577 | 0.254 |
| FRS | 2.007 | 1.344 – 2.997 | <0.001*** |
